# Polish Adaptation of the Yale Physical Activity Survey: Measurement Properties

**DOI:** 10.3390/ijerph16132401

**Published:** 2019-07-06

**Authors:** Magdalena Król-Zielińska, Jacek Zieliński, Adam Kantanista, Robert Szeklicki, Wiesław Osiński, Monika Ciekot-Sołtysiak

**Affiliations:** 1 Department of Physical Education and Lifelong Sports, Faculty of Physical Education, Sport and Rehabilitation, Poznan University of Physical Education, 61-871 Poznan, Poland; 2 Department of Athletics, Strength and Conditioning, Faculty of Physical Education, Sport and Rehabilitation, Poznan University of Physical Education, 61-871 Poznan, Poland; 3 Department of Physical Activity Science and Health Promotion, Faculty of Physical Education, Sport and Rehabilitation, Poznan University of Physical Education, 61-871 Poznan, Poland

**Keywords:** reliability, validity, physical activity measurement, questionnaire, older adults, Yale Physical Activity Survey

## Abstract

The aim of this study was to assess the measurement properties of a Polish adaptation of the Yale Physical Activity Survey (YPAS-PL). The Polish cultural adaptation of the YPAS was administered to a group of 104 people aged 65 to 89 years (mean age 72 ± 5). To assess the reliability of the YPAS-PL, a test-retest procedure was applied. Validity was assessed by comparing the results of the YPAS-PL with accelerometery (ActiGraph wGT3X+). The indicators based on the YPAS-PL activities checklist were characterized by high repeatability and had better reliability values than the YPAS-PL activity dimension indices (energy expenditure interclass correlation coefficient (ICC) = 0.81, total time physical activity ICC = 0.86). We noted a significant positive relationship between energy expenditure measured by an accelerometer and the YPAS-PL (*r* = 0.23). We can conclude that the YPAS-PL is an adequate tool for assessing energy expenditure related to physical activity in a Polish population of older adults. We also recommend the cautious and well thought-out use of the YPAS-PL activity dimension indices (summary, vigorous, leisurely walking, moving, standing, and sitting indexes).

## 1. Introduction

Based on a large body of scientific research, the positive influence of physical activity on health is currently undisputed [1,2]. However, research results suggest that the physical activity of older people decreases with age [3]. Old age is a stage of life when a person is exposed to the occurrence of many diseases. Therefore, the relationship between physical activity and improving the health-related quality of life of older people [4], even those struggling with illnesses, is very important. Some authors suggest that the reported low physical activity of older adults may be the effect of imprecise measurement [5,6,7]. Analyzing the methodology of numerous population studies on older people [5,7,8], we noted researchers often used tools that were created for the younger population. These tools often did not consider the types of activities prevalent in older age groups [9,10].

Among the questionnaires estimating the physical activity of older people, few fulfil the criteria to examine human behavior [5,7,8]. From the reviewed tools, one of the best in terms of formal criteria was the Yale Physical Activity Survey (YPAS) [8]. The YPAS [6] was designed for epidemiological research to measure the level of physical activity during a typical week in the month preceding the study. The YPAS questionnaire was characterized by high indicators of reliability and validity. This tool had high test-retest reliability after 3–5 days [11], and after 2 weeks [6,9,12,13,14]. The validity of the questionnaire was confirmed by correlating the results with accelerometery [6,9,11,12,15,16], Mini-Logger recording [17], double-labelled water method [18,19,20], calorimetry [21,22], VO_2_max [6,23], fitness tests [11,24], body fat [6,9], and other physical activity questionnaires [23].

There is a lack of reliability and validity values of questionnaires used in the studies assessing physical activity levels in older adults in Poland [8]. Kantanista et al. [25] noted that Polish epidemiology studies had used self-reported methods that did not meet psychometric standards, so no reliable data was available on the physical activity of older adults. This limits comparative analyses with other populations. Addressing this issue with ad hoc surveys of physical activity without controlling the criteria that are obligatory for such methods prevents legitimate conclusions. It is essential that physical activity evaluation of the Polish population is monitored in order to conduct research on the determinants of undertaking physical activity of older adults and to create theoretical assumptions of intended interventions aimed at promoting healthy lifestyles.

In view of the above arguments, we decided to adapt the YPAS to the Polish culture, which measures physical activity levels among older adults. The YPAS is available in the English [6], Portuguese [12] and Spanish [9] languages. The intention of the Polish adaptation of the YPAS was to develop a questionnaire to allow for cross-cultural studies and comparisons.

The aim of the present study was to assess the measurement properties of a Polish adaptation of the YPAS. It was hypothesized that a Polish version of the YPAS would have similar reliability and validity values as its original versions and be an accurate questionnaire of assessment of physical activity in the Polish population of older adults.

## 2. Materials and Methods

### 2.1. Procedure

Cultural equivalence refers to the compatibility of theories, dimensions, notions, behavioral indicators, and test procedures. The cultural equivalence criteria of adapted questionnaires consist of item equivalence (the form of the questionnaire), psychometric equivalence (the goodness of test values), functional equivalence (the aim of the assessment), and fidelity of translation and reconstruction [26].

In order to maintain equivalence of the Polish version to the original version of YPAS, a standardized translation procedure was used [27]. Two specialists in the field of physical activity methodology analyzed the theoretical background of the questionnaire with consideration for the differences in the Polish culture and the questionnaire language. They considered that the construct of physical activity used in the questionnaire was culturally appropriate for the Polish community. No words or phrases that are unique to American culture have been found. Two independent bilingual linguists translated the questionnaire into Polish. Two versions of the Polish adaptation of the YPAS questionnaire were created (YPAS-PL(Yale Physical Activity Survey—Polish version)-1 and YPAS-PL-2). Then the physical activity methodology specialists compared and discussed these two versions and agreed on a unified draft version (YPAS-PL-12), which two other bilingual linguists then back-translated. These versions were also compared with the original YPAS questionnaire and discussed. Finally, a Polish version of the YPAS (YPAS-PL) was formed (see Supplementary Table S1). In the Polish language version, racquetball was replaced with badminton in the recreational activities section since badminton is more popular in Poland.

As the next step, the Polish version of the questionnaire was tested for quantitative measurement properties. Potential subjects were informed about the research using the local press, organizations for seniors, leaflets in places frequently visited by older people, and the Poznan University of Physical Education (Poland) website. The participants were informed in detail about the goals and the testing procedure, after which they provided written consent to take part in the project. In accordance with recommendations, a time stability assessment was conducted in the spring. The respondents were familiarized with the questionnaire and given detailed instructions on correct completion. Participants had unlimited time to answer YPAS-PL questions which they completed with the help of a trained interviewer. After completing the questionnaire, participants’ height and weight were measured (digital stadiometer SECA 285, SECA, Hamburg, Germany). The researcher programmed accelerometers ActiGraph model wGT3X+ (ActiGraph, LLC, Pensacola, FL, USA) to record data in epochs of 10 s for 7 consecutive days using the ActiLife6 Analysis Software Suite (ActiGraph, LLC, Pensacola, FL, USA) for analysis. The accelerometer was placed on the anterior superior iliac spine using a belt around the waist [28]. The respondents were trained how to put the device on precisely, and they were instructed to put on the belt in the morning after waking up and to take it off just before bedtime (except bathing, showering, and swimming). After a period of 7 days, the subjects returned the accelerometer and completed the YPAS-PL questionnaire for the second time. The ethics committee at Poznan University of Medical Sciences approved the study (971/12).

### 2.2. Yale Physical Activity Survey

The YPAS was designed for epidemiological research to measure the time devoted to physical activity, energy expenditure, and activity indicators expressed on a point scale. The YPAS-PL assesses physical activity during housework, yardwork, caretaking, exercise, and recreational activities. The recall period applies to a typical week in the month preceding the study. The purpose of the questionnaire is to assess differences between individuals or groups (discrimination). The target population consisted of healthy older adults aged 60 years and over. The questionnaire consists of two sections. The first part is a list of 27 activities, specific for older adults, grouped into five categories (housework, yardwork, caretaking, exercise, and recreational activities). Respondents answer questions on whether they performed (and if so, how much time they spent performing) certain activities from the list during the average week. An index of time devoted to physical activity expressed in hours per week is calculated by summing the time of all activities. The energy expenditure (kcal/week) is estimated by summing the time of each activity multiplied by the corresponding intensity code. The second part (9 questions) is used to assess the level of participation in various types of physical activity. Indicators of five types of activities are estimated by multiplying the duration of each of the following: (a) vigorous activity, (b) leisurely walking, (c) moving, (d) standing, and (e) sitting by an assigned weighting factor. Those indicators are summed to determine the YPAS summary index. The duration of the test is approximately 20 min. The questions used in the first part of the questionnaire are short and do not contain difficult or controversial terminology. The second part contains questions about vigorous physical activity or leisurely walking with a clear definition for each activity.

### 2.3. Reliability

To assess the reliability of the indicators of the YPAS-PL, a test-retest procedure with a one-week interval was applied. Interclass correlation coefficients (ICCs) were calculated using PASW v.18.0 (IBM Corp., Armonk, NY, USA). We assumed the physical activity questionnaire was reliable if the ICC value was above 0.70 (minimum 0.50). The significance level was set at *p* < 0.05 [29].

### 2.4. Validity

The validity was assessed by comparing the results of the YPAS-PL (retest) with accelerometry. The weekly energy expenditure, step counts, minutes per week spent in sedentary, light, moderate, and vigorous activity obtained from the accelerometer were calculated using Freedson’s equation [30] using the ActiLife6 Analysis Software Suite.

Pearson’s correlations for validity were calculated. The significance level for the Pearson correlation coefficient was set at *p* < 0.05. Similar to Terwee et al. [29], we assumed the physical activity questionnaire was valid if the correlation was above 0.50 for the accelerometer. Statistical analyses were performed using STATISTICA 13 software (StatSoft, Inc., Tulsa, OK, USA).

### 2.5. Participants

One hundred and four older adults (75 women and 29 men) participated in this study. None of the respondents was excluded from the final analysis (individuals with missing data were asked to complete all procedures one week later). Respondents were people aged 65 to 89 (mean age 72 ± 5 years). Most were married (53%), and 31% were widows or widowers. In terms of education, 45% of the respondents graduated from university, 34% secondary school, 13% vocational school, and 8% primary school. The average body height of the women was 1.59 ± 0.06 m, body weight was 67.8 ± 12.6 kg, and Body Mass Index (BMI) was 26.7 ± 4.0 kg/m^2^. In the men, the average body height was 1.70 ± 0.06 m, body weight was 80.3 ± 9.6 kg, and BMI was 27.8 ± 3.6 kg/m^2^.

## 3. Results

### 3.1. Reliability

The test-retest results of the YPAS-PL are presented in Table 1. Acceptable ICC values have been achieved for energy expenditure (ICC = 0.86), total time (ICC = 0.81), and leisurely walking index (ICC = 0.78). Three of the eight indicators in the YPAS-PL questionnaire had reliability parameters below 0.50 (vigorous activities index, standing index, and sitting index).

### 3.2. Validity

We noted a positive relationship between caloric expenditure measured by the accelerometer and YPAS-PL energy expenditure (*r* = 0.23). The accelerometer time in moderate (but no time in vigorous and light) activity correlated favorably with the energy expenditure YPAS-PL parameter (*r* = 0.23). We observed an inverse association for time in sedentary activity measured by the accelerometer and YPAS-PL vigorous index (*r* = −0.27). None of the correlation coefficients between the YPAS-PL indicators and the accelerometer data reached the assumed minimum for study validity according to Terwee et al. [29] (above 0.50, see Table 2).

## 4. Discussion

This study examined the measurement properties of a Polish adaptation of the YPAS. The aim of the translation and validation study of the questionnaire was to assess the possibility of using the questionnaire in Poland and to compare with international data.

In our adaptation, a translation strategy was decided because it was assumed that the construct of physical activity used in the YPAS questionnaire could be considered culturally appropriate for the Polish community. The second reason was the desire to create an adaptation of the questionnaire, which will allow for cross-cultural studies and comparisons.

The main finding of this study was that the indicators based on the YPAS-PL activities checklist (energy expenditure and total time physical activity) had better reliability values than the YPAS-PL activity dimension indices (in particular vigorous, standing and sitting indexes). The YPAS-PL energy expenditure and total time physical activity has acceptable validity parameters. The one-week test-retest results ranged from 0.40 to 0.86, suggesting good reliability for some YPAS-PL indicators but insufficient for others. In our study, optimum repeatability rates were obtained in energy expenditure (ICC = 0.81), total time physical activity (ICC = 0.86), leisurely walking index (ICC = 0.78), and summary index (ICC = 0.69), but the standing, sitting, and vigorous activities indices had reliability parameters below the minimum of 0.50. Also, in the Spanish version of the YPAS (YPAS-ESP) [9], total time and energy expenditure repeated better (ICC = 0.66 and ICC = 0.65, respectively) than the YPAS-ESP activity dimension indices (ICC = 0.12–0.33). Similar reliability coefficients were obtained for energy expenditure (ICC = 0.92) and total time physical activity (ICC = 0.92) in the Portuguese adaptation of the YPAS (YPAS-PT) after two weeks test-retest [12].

The differences between the reliability results of the indicators based on the YPAS-PL activity checklist (energy expenditure and total time physical activity) and YPAS-PL activity dimension indices (especially vigorous, standing and sitting indexes) may be due to the questionnaire design. In our study, several respondents reported difficulties in understanding the questions about intensity of physical activity contained in the second part of the questionnaires. Other authors also noted problems in the participants’ interpretation of questions regarding the intensity of physical activity. For example, Altschuler et al. [31] noticed that respondents might interpret the intensity of activity in different ways. Moreover, other studies suggested that people tended to overestimate the subjective assessment of perceived exertion [32,33]. The low reliability of vigorous index may be caused by a different perception of exercise intensity by the same person on different days. This may depend on many factors (e.g., current fatigue, temper, drugs, etc.). The low repeatability of standing and sitting indexes may be caused by difficulties in estimating the proper time spending in those positions. Respondents had to answer how many hours they “spend on standing/sitting on an average day during the past month”.

We hypothesized that the YPAS-PL would have similar reliability values as its original version. The test-retest correlations in the first validation study of the YPAS [6] were 0.65 for summary index, 0.61 for vigorous index, 0.58 for energy expenditure, 0.57 for total time physical activity, and below 0.50 for all other indices. In the study by DiPietro et al. [6], reliability was assessed by Pearson correlation coefficients (two-week interval). The specialists in the field of physical activity methodology suggested that the most adequate reliability parameter is the ICC, as the Pearson correlation does not take into account systematic differences between the two measurements [29]. They argued that the Pearson correlation coefficients in reliability studies might overestimate results [29], which is why the repeatability of the original YPAS was questioned.

Pennathur et al. [18] tested the reliability of the YPAS with older Mexican American adults. The ICCs for almost all parameters based on the YPAS were below 0.50. In Schuler et al.’s [14] study, similar to our results, total time physical activity (ICC = 0.74) and energy expenditure (ICC = 0.74) repeated much better than the activity dimension indices (summary index ICC = 0.55, vigorous index ICC = 0.48, leisurely walking index ICC = 0.41, moving index ICC = 0.60, standing index ICC = 0.22, sitting index ICC = 0.13). To summarize, indicators based on the YPAS-PL activities checklist were characterized by high repeatability and performed similarly in terms of reliability in comparison with previous studies.

We evaluated the validity of the YPAS-PL by correlating the results with the accelerometer. We assumed (after Terwee et al. [29]) that the YPAS-PL is valid if the correlation coefficients for the accelerometer and YPAS-PL results were above 0.50. None of the validity results of the YPAS-PL indicators reached this minimum value. Other authors interpreted the strength of validity using Cohen’s classification [34]. According to this classification, the values of the correlation coefficients may be small (0.10), medium (0.30), and large (0.50). Based on this assumption, the relationship between caloric expenditure measured by the accelerometer and YPAS-PL energy expenditure was small (*r* = 0.23). Low correlation was observed for the accelerometer time in moderate activity and YPAS-PL energy expenditure (*r* = 0.23). We noted an inverse small correlation for time in the sedentary measured by the accelerometer and YPAS-PL vigorous index (*r* = −0.27). Only these three validation coefficients of the YPAS-PL and accelerometer data were statistically significant.

Our validity results are in some part comparable with the results of DiPietro et al. [6]. The parameters of the original version of the YPAS showed no significant correlation with all accelerometer data. The best YPAS validity results have been achieved in the Spanish version of the YPAS [15]. Correlation coefficients between the questionnaire and accelerometer data showed medium to large (*r* = 0.32–0.52) validity of the YPAS-ESP. Donaire-Gonzalez et al. [15] reached higher correlation between energy expenditure measured by the accelerometer and self-reported energy expenditure (*r* = 0.37) than in our research (*r* = 0.23). In another validation of the YPAS-ESP [9], total time, energy expenditure, summary index, leisurely walking index, and moving index correlated significantly with the accelerometer (*r* = 0.20, *r* = 0.23, *r* = 0.24, *r* = 0.26, and *r* = 0.31, respectively). The same low correlation (*r* = 0.23) was observed for the YPAS-PL energy expenditure and accelerometer; however, in our study, no other accelerometer parameters correlated with YPAS-PL activity dimension indices. Machado et al. [12] found medium correlation between total time in physical activity between the YPAS-PT and accelerometer (*r* = 0.41). However, the YPAS-PT indices showed no significant correlation with energy expenditure measured by the accelerometer. We also noted an inverse correlation for time in the sedentary measured by the accelerometer and YPAS-PL vigorous index (*r* = −0.27). A similar pattern of association was reported in the YPAS-PT validation studies [12]. The YPAS-PT sitting index was related to the accelerometer time in vigorous activity (*r* = 0.31).

Machado et al. [12] suggested the accelerometer data might not be sensitive within older people. They explained that cutoff points based on Freedson’s equation [30] might be set too high for measuring low-intensity activities that characterize older adults. Other authors have suggested that an accelerometer worn on the hip may not adequately estimate the low-intensity physical activity associated with arm movement [35]. Talbot et al. [36] noted that older adults were characterized by mainly low-intensity activities. This observation was also noticed in older adults in Poland [37].

Older adults in our research were asked to take off the accelerometer during activities in the water, including swimming and water gymnastics. Considering that our respondents showed participation in water activities, we are of the opinion that it could have influenced the results of validity indicators with the accelerometer. Physical activity performed in water affected the results of YPAS-PL. At the same time, water activities were not counted by the accelerometer. This could have decreased correlation coefficients between physical activity measured by the accelerometer and the YPAS-PL.

The YPAS-PL validity results for energy expenditure were similar to validity studies of the original version of the survey as well as the Spanish and Portuguese translations. For other indices based on the YPAS-PL, the dependency pattern did not coincide with the results of previous studies. The above considerations indicate that the YPAS-PL is an adequate tool for assessing energy expenditure. Our adaptation of the YPAS can be used for cross-cultural studies and comparisons, especially in the case of energy expenditure. The appropriate assessment of the energy expenditure of older people will be extremely helpful in creating the theoretical assumptions of intended interventions aimed at promoting healthy lifestyles in Poland. A reliable diagnosis of the initial state of the energy expenditure of older adults will allow the proper selection of training loads in order to raise or maintain physical activity at an appropriate level. An analysis and comparison of the final state will be helpful in assessing the effectiveness of physical activity programs in this age group.

Based on our results, we can partially confirm the hypothesis regarding the similarities of reliability and validity values between the Polish adaptation of the YPAS and its original version and the YPAS-PL is an accurate questionnaire for the assessment of some parameters of physical activity (energy expenditure) in the Polish population of older adults.

## 5. Conclusions

We can conclude that the YPAS-PL is an adequate tool for assessing energy expenditure related to physical activity in a Polish population of older adults. We also recommend the cautious and well thought-out use of the YPAS-PL activity dimension indices (summary, vigorous, leisurely walking, moving, standing, and sitting indexes).

## Figures and Tables

**Table 1:** Test-Retest Results of the YPAS-PL—Descriptive Statistics and ICC.

YPAS-PL	Test Mean ± SD	Retest Mean ± SD	ICC
Energy expenditure (kcal per week)	7106.0 ± 4749.9	6587.7 ± 4262.2	0.81 **
Total time PA (hours per week)	30.7 ± 18.8	28.8 ± 17.3	0.86 **
Summary index (total units)	50.2 ± 30.3	53.3 ± 34.6	0.69 **
Vigorous index (units·month^−1^)	15.1 ± 21.3	17.0 ± 23.5	0.40 **
Leisurely walking index (units·month^−1^)	17.6 ± 16.3	19.4 ± 16.6	0.78 **
Moving index (hours·day^−1^)	10.7 ± 2.7	10.3 ± 2.9	0.63 **
Standing index (hours·day^−1^)	4.8 ± 2.5	4.5 ± 2.3	0.48 **
Sitting index (hours·day^−1^)	1.9 ± 0.8	2.1 ± 1.0	0.45 **

ICC—Interclass correlation coefficient, PA—physical activity, SD—standard deviation, YPAS-PL—Polish adaptation of the Yale Physical Activity Questionnaire, ** *p* < 0.01.

**Table 2:** Correlation Coefficients between Parameters of Physical Activity Measured by the YPAS-PL and Accelerometer.

YPAS-PL	Accelerometer					
	Energy Expenditure	Time in Vigorous Activity	Time in Moderate Activity	Time in Light Activity	Time in Sedentary	Steps Counts
Energy expenditure	0.23 *	0.02	0.23 *	−0.01	0.10	0.17
Total time PA	0.16	0.01	0.19	−0.02	0.12	0.14
Summary index	−0.08	−0.07	0.07	−0.12	−0.21	0.05
Vigorous index	−0.08	−0.1	0.03	−0.06	−0.27 *	0.02
Leisurely walking index	−0.05	−0.01	0.08	−0.15	0.03	0.06
Moving index	0.14	0.02	0.10	0.01	0.01	0.08
Standing index	0.04	0.03	0.04	−0.05	0.03	−0.01
Sitting index	0.03	−0.09	0.05	−0.06	0.07	−0.07

PA—physical activity, YPAS-PL—Polish adaptation of the Yale Physical Activity Questionnaire, * *p* < 0.05.

## Supplementary Materials

The following are available online at https://www.mdpi.com/1660-4601/16/13/2401/s1, Table S1: Kwestionariusz aktywności fizycznej dla osób starszych (YPAS-PL).
