# Supplementary material for: Polish Adaptation of the Yale Physical Activity Survey: Measurement Properties
Source: Int J Environ Res Public Health. 2019 Jul 6;16(13):2401. doi: 10.3390/ijerph16132401 (PMC6651623; doi:10.3390/ijerph16132401)
Supplement: Supplementary file 1 [file ijerph-16-02401-s001.pdf]

KWESTIONARIUSZ AKTYWNOŚCI FIZYCZNEJ
DLA OSÓB STARSZYCH (YPAS-PL)

PROSZĘ PODAĆ CZAS: ________:________:________
godz. min s

Oto lista powszechnie podejmowanych rodzajów aktywności fizycznej. Proszę wskazać, które spośród nich wykonywał/a Pan/i podczas typowego tygodnia w ostatnim miesiącu. Chcielibyśmy zapoznać się z rodzajami aktywności fizycznej, które wykonuje Pan/i w czasie zwyczajowej pracy i w czasie wolnym.

Dla każdej wykonywanej czynności, proszę podać ile czasu (w godzinach) poświęcił/a Pan/i na nią w ciągu typowego tygodnia.

**Praca**	**Czas (Godz./tyg.)**	**Kod intensywności (kcal/min)**
Zakupy (np. artykuły spożywcze, ubrania, itd.)		3,5
Wchodzenie po schodach z obciążeniem (np. z zakupami)		8,5
Pranie (jedynie czas przeznaczony na ładowanie i wyjmowanie prania z pralki, wieszanie i składanie prania)		3,0
Lekkie prace domowe: porządkowanie, ścieranie kurzu, zamiatanie; zbieranie śmieci w mieszkaniu; polerowanie mebli; pielęgnowanie roślin doniczkowych; prasowanie		3,0
Ciężkie prace domowe: odkurzanie, mycie podłóg i ścian; przesuwanie mebli, pudeł, pojemników na śmieci		4,5
Przygotowywanie posiłków (powyżej 10 minut): krojenie, mieszanie; przemieszczanie się po produkty i sprzęty		2,5
Podawanie posiłków (powyżej 10 minut): nakrywanie do stołu; przynoszenie i podawanie jedzenia		2,5
Zmywanie naczyń (powyżej 10 minut): sprzątanie ze stołu, mycie/wycieranie naczyń, odkładanie naczyń na miejsce		2,5
Lekkie prace naprawcze: naprawa małych urządzeń; drobne naprawy w domu		3,0
Ciężkie prace naprawcze: malowanie, stolarka, mycie/polerowanie samochodu		5,5
Inne:		+
**Prace na zewnątrz domu, w ogrodzie lub na działce**		
Prace ogrodowe: sadzenie, pielenie, kopanie, gracowanie		4,5
Koszenie trawy (tylko chodzenie z kosiarką)		4,5
Sprzątanie alejek, chodników/podjazdu: zamiatanie, odgarnianie, grabienie		5,0
Inne:		+
**Opieka**		
Opieka nad osobą starszą lub niepełnosprawną (podnoszenie, pchanie wózka inwalidzkiego)		5,5
Opieka nad dzieckiem (podnoszenie, noszenie, pchanie wózka)		4,0
Inne:		+

Ćwiczenia		
Szybki marsz (powyżej 10 minut)		6,0
Ćwiczenie w basenie, rozciąganie, joga		3,0
Intensywne ćwiczenia gimnastyczne, aerobik		6,0
Jazda na rowerze lub na rowerze stacjonarnym		6,0
Pływanie (pokonywanie dystansu)		6,0
Inne:		+

Aktywność w czasie wolnym		
Rekreacyjny spacer (powyżej 10 minut)		3,5
Robótki ręczne: robienie na drutach, szycie, haftowanie, itd.		1,5
Taniec: w parach, tańce grupowe, itd.		5,5
Kręgle, bule, boccia, itd.		3,0
Golf (tylko chodzenie od dołka do dołka)		5,0
Tenis, squash, badminton, itd.		7,0
Bilard		2,5
Inne:		+

Chcę teraz zapytać o wybrane formy aktywności fizycznej wykonywanej przez Pana/Panią w ciągu ostatniego miesiąca. Ile czasu poświęcał/a Pan/i na aktywność fizyczną o wysokiej intensywności, spacerowanie w wolnym tempie, przebywanie w pozycji siedzącej i stojącej oraz inne czynności, które zwykle Pan/i wykonuje.

1. Mniej więcej ile razy w ciągu miesiąca podejmował/a Pan/i aktywność fizyczną o wysokiej intensywności, która trwała powyżej 10 minut i wywoływała znaczne przyspieszenie oddechu, akcji serca oraz zmęczenie nóg lub pocenie się?

- ☐ Wcale
- ☐ 1-3 razy w miesiącu
- ☐ 1-2 razy w tygodniu
- ☐ 3-4 razy w tygodniu
- ☐ 5 i więcej razy w tygodniu
- ☐ Nie wiem

2. Mniej więcej jak długo jednorazowo była wykonywana taka intensywna aktywność fizyczna?

- ☐ Wcale
- ☐ 10-30 minut
- ☐ 31-60 minut
- ☐ Ponad 60 minut
- ☐ Nie wiem

3. Proszę pomyśleć o spacerach, na których Pan/Pani był/a w ostatnim miesiącu. Mniej więcej ile razy w ciągu ostatniego miesiąca spacerował/a Pan/Pani przynajmniej 10 minut lub więcej bez zatrzymywania się w tempie, które nie spowodowało znacznego przyspieszenia oddechu, akcji serca oraz zmęczenie nóg lub spocenia się?

- ☐ Wcale
- ☐ 1-3 razy w miesiącu
- ☐ 1-2 razy w tygodniu
- ☐ 3-4 razy w tygodniu
- ☐ 5 i więcej razy w tygodniu
- ☐ Nie wiem

4. Ile minut trwały te spacery?

- ☐ Wcale
- ☐ 10-30 minut
- ☐ 31-60 minut
- ☐ Ponad 60 minut
- ☐ Nie wiem

5. Mniej więcej ile godzin w ciągu dnia porusza się Pan/i chodząc podczas wykonywania różnych czynności? Proszę podać jedynie czas, kiedy jest Pan/i faktycznie w ruchu.

- ☐ Wcale
- ☐ mniej niż 1 godzinę dziennie
- ☐ 1 do mniej niż 3 godziny dziennie
- ☐ 3 do mniej niż 5 godziny dziennie
- ☐ 5 do mniej niż 7 godzin dziennie
- ☐ 7 i więcej godzin dziennie
- ☐ Nie wiem

6. Proszę pomyśleć ile czasu spędził/a Pan/i stojąc w ciągu przeciętnego dnia podczas ostatniego miesiąca. Ile godzin dziennie spędza Pan/i w pozycji stojącej?

- ☐ Wcale
- ☐ mniej niż 1 godzinę dziennie
- ☐ 1 do mniej niż 3 godziny dziennie
- ☐ 3 do mniej niż 5 godziny dziennie
- ☐ 5 do mniej niż 7 godzin dziennie
- ☐ 7 i więcej godzin dziennie
- ☐ Nie wiem

7. Mniej więcej ile godzin w ciągu przeciętnego dnia spędził/a Pan/i w pozycji siedzącej podczas ostatniego miesiąca?

- ☐ Wcale
- ☐ mniej niż 3 godziny dziennie
- ☐ 3 do mniej niż 6 godzin dziennie
- ☐ 6 do mniej niż 8 godzin dziennie
- ☐ 8 i więcej godzin dziennie
- ☐ Nie wiem

8. Mniej więcej ile kondygnacji schodów pokonuje Pan/i wchodząc na górę każdego dnia? Przyjmujemy, że 10 schodów to 1 kondygnacja.

____

9. Proszę porównać poziom aktywności fizycznej podejmowanej przez Pana/Panią w innych porach roku z poziomem aktywności fizycznej, który właśnie Pan/i określił/a w odniesieniu do typowego tygodnia podczas ostatniego miesiąca. Na przykład, czy w miesiącach letnich podejmuje Pan/i więcej czy mniej aktywności fizycznej niż w ciągu ostatniego miesiąca?

	**Dużo więcej**	**Trochę więcej**	**Tak samo**	**Trochę mniej**	**Dużo mniej**	**Nie wiem**
Wiosna	1.30	1.15	1.00	0.85	0.70	-
Lato	1.30	1.15	1.00	0.85	0.70	-
Jesień	1.30	1.15	1.00	0.85	0.70	-
Zima	1.30	1.15	1.00	0.85	0.70	-
